# The way from pen and paper to electronic documentation in a German emergency department

**DOI:** 10.1186/s12913-019-4400-y

**Published:** 2019-08-09

**Authors:** Benjamin Lucas, Peter Schladitz, Wiebke Schirrmeister, Gerald Pliske, Felix Walcher, Martin Kulla, Dominik Brammen

**Affiliations:** 1 Department of Trauma Surgery, Otto-von-Guericke University Magdeburg, D 39120 Magdeburg, Germany; 2 Department of Anaesthesiology, Intensive Care Medicine, Emergency Medicine and Pain Therapy, Bundeswehrhospital Ulm, Oberer Eselsberg 40, 89081 Ulm, Germany; 3 Department of Anaesthesiology and Intensive Care, Otto-von-Guericke University Magdeburg, D-39120 Magdeburg, Germany

**Keywords:** Emergency department, Electronic health records, Registry

## Abstract

**Background:**

Some of the advantages of implementing electronic emergency department information systems (EDIS) are improvements in data availability and simplification of statistical evaluations of emergency department (ED) treatments. However, for multi-center evaluations, standardized documentation is necessary. The AKTIN project (“National Emergency Department Register: Improvement of Health Services Research in Acute Medicine in Germany”) has used the “German Emergency Department Medical Record” (GEDMR) published by the German Interdisciplinary Association of Intensive and Emergency Care as the documentation standard for its national data registry.

**Methods:**

Until March 2016 the documentation standard in ED was the pen-and-paper version of the GEDMR. In April 2016 we implemented the GEDMR in a timeline-based EDIS. Related to this, we compared the availability of structured treatment information of traumatological patients between pen-and-paper-based and electronic documentation, with special focus on the treatment time.

**Results:**

All 796 data fields of the 6 modules (basic data, severe trauma, patient surveillance, anesthesia, council, neurology) were adapted for use with the existing EDIS configuration by a physician working regularly in the ED. Electronic implementation increased availability of structured anamnesis and treatment information. However, treatment time was increased in electronic documentation both immediately (2:12 ± 0:04 h; *n* = 2907) and 6 months after implementation (2:18 ± 0:03 h; *n* = 4778) compared to the pen-and-paper group (1:43 ± 0:02 h; *n* = 2523; *p* < 0.001).

**Conclusions:**

We successfully implemented standardized documentation in an EDIS. The availability of structured treatment information was improved, but treatment time was also increased. Thus, further work is necessary to improve input time.

## Background

There are several good reasons for implementing electronic emergency department information systems (EDIS), including the reduction of medical errors and improvements in treatment quality [1]. Presently, information systems can be based on one of several different technical designs. It is possible to implement an EDIS by having commercial providers supply either specialized emergency department (ED) modules for a current hospital information system (HIS) or highly customized stand-alone systems (“best of breed”). On the other hand, an existing system in a hospital can be adapted for use in the ED via proprietary development.

Regardless of the chosen approach, it is important to tailor the EDIS to individual workflow and documentation requirements. In Germany, the current documentation standard for EDs is the “German Emergency Department Medical Record” (current version V2015.1), which was first published in 2010 by the German Interdisciplinary Association of Intensive and Emergency Care (DIVI) [2–4]. The German Emergency Department Medical Record (GEDMR) was utilized in the HL7 Clinical Document Architecture (HL7-CDA), which is used as an interface for a national ED data registry prepared by the joint research project “National Emergency Department Register: Improvement of Health Services Research in Acute Medicine in Germany” (AKTIN) [3, 5, 6].

Klinger et al. have already implemented this medical record standard successfully using digital pen-and-paper technology [7]. However, the main disadvantage of their solution is that it was not connected with patient data management systems (PDMS).

We integrated the complete GEDMR into an existing PDMS in the ED of a Level 1 Trauma Center in order to help collect data for the national ED data registry. Previous to the electronic implementation, the pen-and-paper version 2015.1 of the GEDMR was used in this ED [2–4].

Here, we compare the documentation compliance of pen-and-paper based ED documentation with electronic implementation in a Level 1 Trauma Center, with special focus on influence on treatment time. Therefore, we analyzed the usage frequency of each documentation field containing structured information, like tetanus or pregnancy. For treatment time analysis, the time gap between admission and discharge was evaluated.

## Methods

In this study we collected data retrospectively and monocentrically. The data were anonymized for the personal data of the patients and the documenting physician. For analysis, we divided the data into 3 time periods. The “pen-and-paper” period was from October 2015 to March 2016. The implementation of the GEDMR was executed in April 2016, and so the “post-implementation” period was from April 2016 to September 2016. Finally, data from October 2016 to August 2017 were grouped in the “regular-use” period to acquire possible training effects in comparison to the first 6 month of use in the post-implementation period. The methodology to report this study adheres to the STrengthening the Reporting of OBservational studies in Epidemiology (STROBE) statement [8].

### German emergency department medical record

The currently available GEDMR is version V2015.1, which comprises 796 data items [2, 4, 6]. It has been approved by the German DIVI. From its creation, the developers focused on ensuring an interdisciplinary and interprofessional approach meeting the requirements of any type of specialist working in the ED [4]. Originally, the medical record had a modular structure [6]. The basic module contained fields for documenting standard information of each patient, such as anamnesis, treatment and procedure. The extended modules contained fields for documenting special cases, such as seriously injured patients or monitored patients [6].

### Pen-and-paper-based documentation

Pen-and-paper documentation was based on the paper GEDMR Version 2015.1 [2–4]. For evaluation sections, the GEDMR was grouped into field groups. Traumatological documentation of a Level 1 Trauma Center for any traumatological patient who was not treated in a trauma room from October 2015 to March 2016 was collected. Patients insured by the German Social Accident Insurance were excluded, because of differing documentation protocols/requirements [9].

### Electronic documentation “ICUdata”

In the ED of the University of Magdeburg, a PDMS called “ICUdata” (IMESO, Gießen, Germany) has been used since 2012. This system, originally designed for use in intensive care units (ICUs), has been adapted with a unique configuration for use in the ED. In 2002, ICUdata was installed at an anesthesiology ICU. Thereafter, it was extended to 4 other ICUs, including a further anesthesiology ICU, an intermediate care unit, a stroke unit, and two neurological wards. ICUdata [10] is a multi-server, multi-client system, meaning that the system load is distributed over a server cluster. It allows for case documentation for the same patient to be accessed from several client workstations, and makes it possible to open documentation for multiple patients simultaneously. Vital records can be transferred automatically using an RS-232 interface or a central HL7 gateway. Patient master data (i.e., Admission, Discharge, Transfer) and laboratory data are received by a communication server, which transfers this information from the HIS and laboratory information system to a mirror database in the PDMS. Images from radiological diagnostic testing results can be opened in a web-based viewer via a context-based call directly in ICUdata. The basic structure of the system divides data into a number of categories, including patient data, examinations and results, interventions, care, notes, drug applications, liquid balance, vital parameters, respiratory data, and laboratory data. The dialog windows for data input are freely configurable.

However, to integrate the medical record into the structure of the PDMS in December 2015, the structure of the GEDMR was made holistic, such that all data items were integrated as a single pool in the PDMS. For structural documentation of presenting complaints, we integrated the German translation of the Canadian Emergency Department Information System (CEDIS) list, which contains 169 items and 2 special presenting problems [11, 12], as it was required by the AKTIN project. The items of the translated CEDIS list were grouped in 17 categories for a faster approach similar to the original layout [13]. This enables the structural analysis of treatment cases relating to a specific presenting complaint and benchmarking of the ED [14]. The medical record and the CEDIS list were implemented by a physician regularly working in the ED. The PDMS was mandatory for traumatological patients in the ED beginning in April 2016; before, it was possible to use the PDMS optionally. From April 2016 to August 2017 treatment data corresponding to the pen-and-paper-based documentation was collected by electronically accessing the PDMS database, directly mapping the data to the field groups of the pen-and-paper-based documentation. Moreover, patients that were not documented electronically were included with basic information, such as gender, age, and admission time. For statistical analyses, we divided the data into a “post-implementation” group (April 2016 to September 2016) and a “regular-use” group (October 2016 to August 2017).

### Statistical analyses

For comparison of treatment time we used the time gap between administrative admission and discharge from ED. Documentation compliance was compared by analyzing the frequency of filled documentation field groups with structured information (allergies, main diagnosis, diagnostic, referrer, vaccination status against tetanus, transport vehicle, presenting complaints, and discharge).

For statistical analyses SPSS Statistics 24 (IBM, Armonk, USA) was used. All data are presented as relative frequencies or mean ± standard error of means (SEM). Descriptive statistics using the Chi-Squared test was performed for categorical data. For treatment time we used the Kruskal–Wallis test because of the non-normal distribution of the data (Kolmogorov–Smirnov *p* < 0.001). *P*-values lower than 0.05 were considered statistically significant. For post-hoc pairwise tests the significance levels were adjusted for multiple testing using the Bonferroni correction.

## Results

**Fig. 1:**
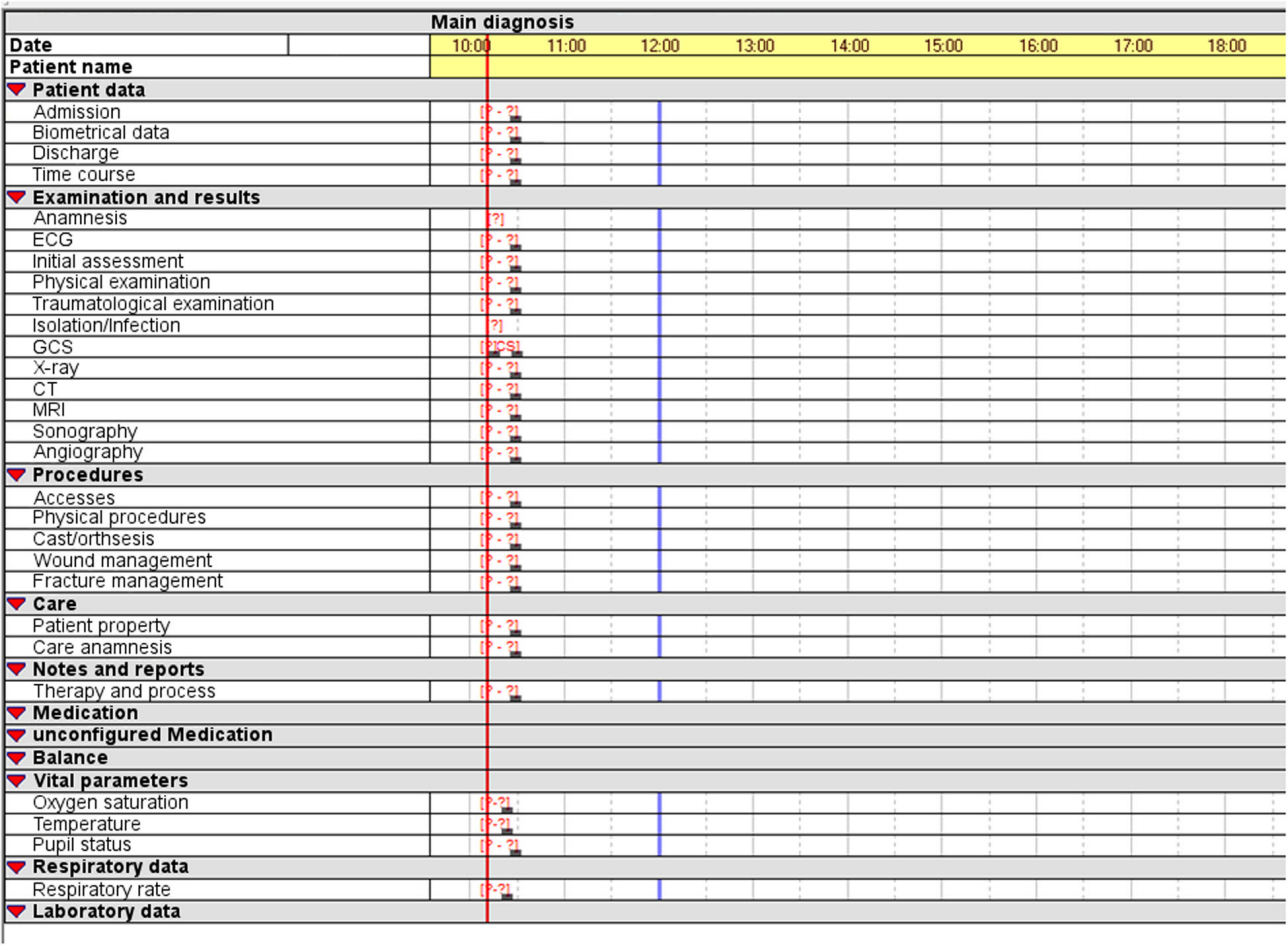
Depiction of the implemented basic module. Modified screenshot (translated into English) of the electronic patient chart. This is an overview of an electronic patient chart after loading the preset for the complete basic module, which includes each dialog window associated with the basic module of the German Emergency Department Medical Record of the DIVI

All 796 data fields of the 6 modules (basic data, severe trauma, patient surveillance, anesthesia, council, neurology) of the German Emergency Department Medical Record were implemented into the ED’s existing configuration of the PDMS according to the workflow in the ED. As noted above, modularization of the medical record was resolved. The original data fields were distributed among 33 dialog windows; the basic module was distributed among 20 dialog windows, supplemented by eight additional dialog windows, six of which contained data fields for documenting standard procedures and examinations carried out in EDs such as wound management, and two containing data fields for nursing documentation (Fig. 1). All dialog windows could be loaded on demand. This enables the physician to focus on the actual complaints of and diagnostic elements for a particular patient. Furthermore, it was not necessary to code time stamps in the data fields, since the PDMS itself was timeline-based; as such, when any record is saved in the database, so is the input and observation time.

From 10/2015 to 08/2017 17,675 ED documentation of traumatological patients was analyzed.

For the pen-and-paper period (10/2015 to 03/2016) 3962 documents were found, of which 3199 were documented with the pen-and-paper version of the GEDMR V2015.1. Treatment cases documented on paper consisted of 1656 male (average age 44.05 ± 0.52 years) and 1542 female (average age 53.02 ± 0.61 years) patients. For the post-implementation period (04/2016 to 09/2016), 5665 patients were analyzed, with 2910 PDMS-documented cases. The PDMS-documented treatment cases consisted of 1481 male (average age 44.19 ± 0.56 years) and 1429 (average age 54.76 ± 0.63 years) female patients. In the period with regular use (10/2017 to 08/2017) of the PDMS 8048 cases were analyzed, with 4782 PDMS-documented cases, consisting of 2385 male (average age 44.54 ± 0.46 years) and 2397 female (average age 58.15 ± 0.49 years) patients (Tab. 1).

**Table 1:** Average age (years) and sample size in relation to the documentation period and gender

	pen-and-paper (10/2015–03/2016)	post-implementation (04/2016–09/2016)	regular-use (10/2016–08/2017)
male	44.05 ± 0.52 years (*n* = 1656)	44.19 ± 0.56 years (*n* = 1481)	44.54 ± 0.46 years (*n* = 2385)
female	53.02 ± 0.61 years (*n* = 1542)	54.76 ± 0.64 years (*n* = 1429)	58.15 ± 0.49 years (*n* = 2397)

**Fig. 2:**
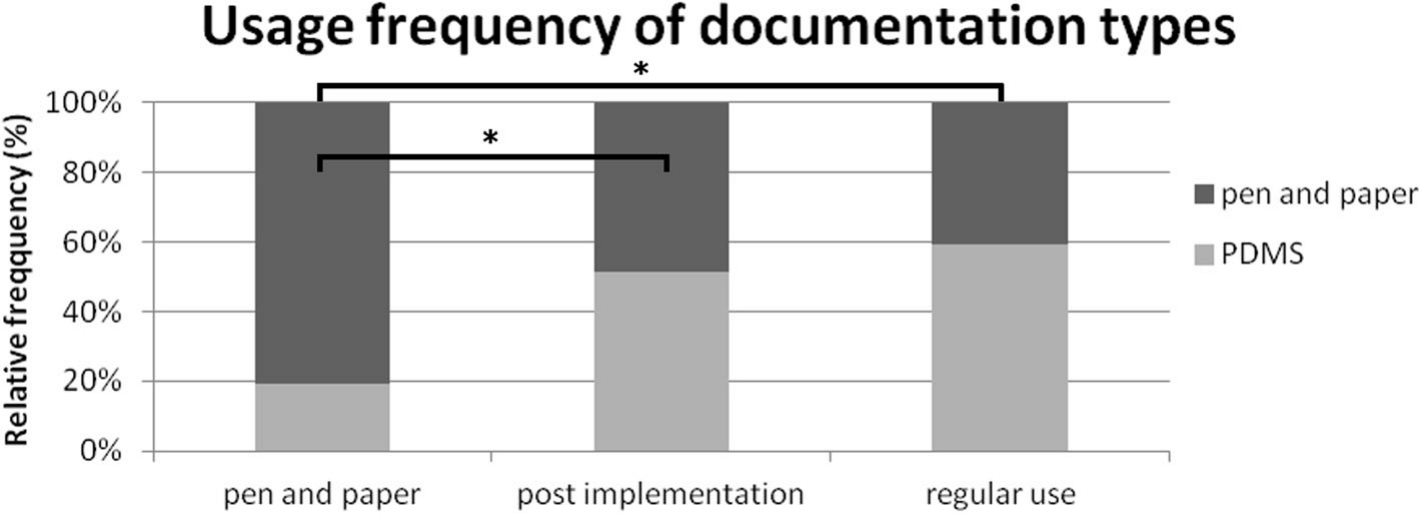
Usage frequency of documentation types. Usage frequency of pen-and-paper and electronic (PDMS) documentation was compared. Usage of the PDMS increased significantly from pen-and-paper (19.3%; *n* = 3962) to post-implementation period (51.4%; *n* = 5665; *p* < 0.05; χ^2^ test) and regular-use period (59.4%; *n* = 8048; *p* < 0.05; χ^2^ test)

From the pen-and-paper period (19.3%) to the post-implementation period (51.4%) we could see a significant increase in the usage frequency of PDMS documentation (Fig. 2; *p* < 0.05). However, from the post-implementation to the regular-use period there was also an increase in usage frequency (p < 0.05), from 51.4 to 59.4%.

For further statistical analyses such as descriptive and interference statistics, only treatment cases that were pen-and-paper documented in the pen-and-paper period or PDMS-documented in post-implementation and regular-use period were used.

**Fig. 3:**
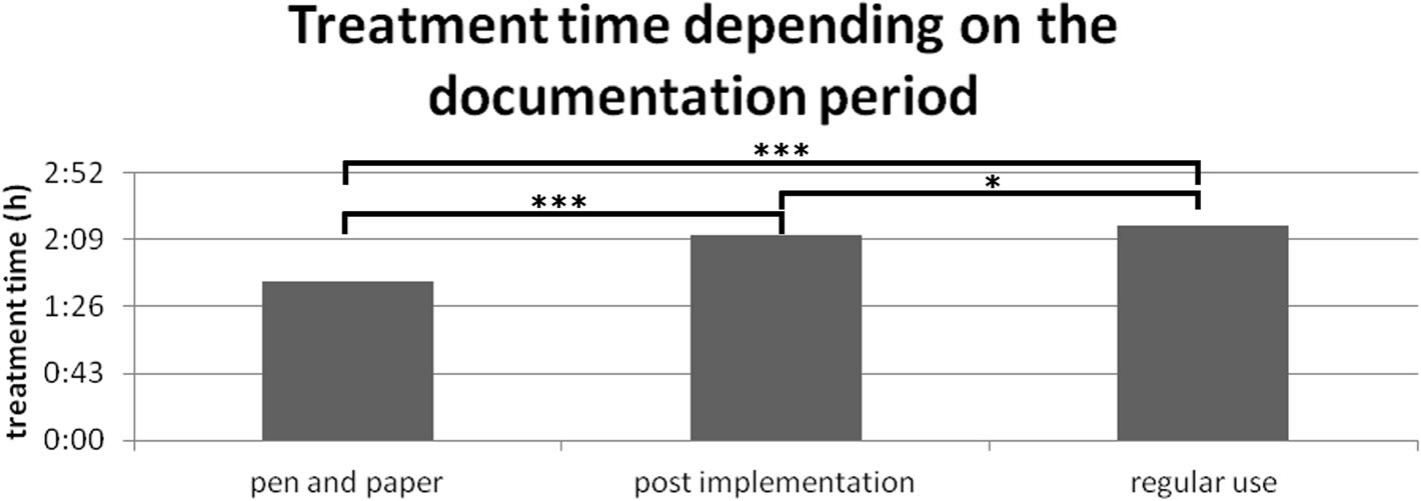
Treatment time depending on the documentation period. Treatment time depending on documentation period: significant increase from pen-and-paper (1:43 ± 0:02 h; *n* = 2523) to post-implementation (2:12 ± 0:04 h; *n* = 2907; *p* < 0.001) and regular-use period (2:18 ± 0:03 h; *n* = 4778; *p* < 0.001). However, we can also show a significant increase in treatment time from post-implementation to regular-use period (*p* = 0.013). Kolmogorov–Smirnov *p* < 0.001; Kruskal–Wallis *p* < 0.001; post-hoc with Bonferroni correction; * *p* < 0.05; *** *p* < 0.001

Relating to treatment times (administrative admission to discharge) a significant increase in the post-implementation (2:12 ± 0:04 h; *n* = 2907) and regular-use group (2:18 ± 0:03 h; *n* = 4778) compared to the pen-and-paper group (1:43 ± 0:02 h; *n* = 2523) was shown (Fig. 3, *p* < 0.001). However, between post-implementation and regular-use period a significant increase in treatment time was shown (*p* = 0.013).

**Fig. 4:**
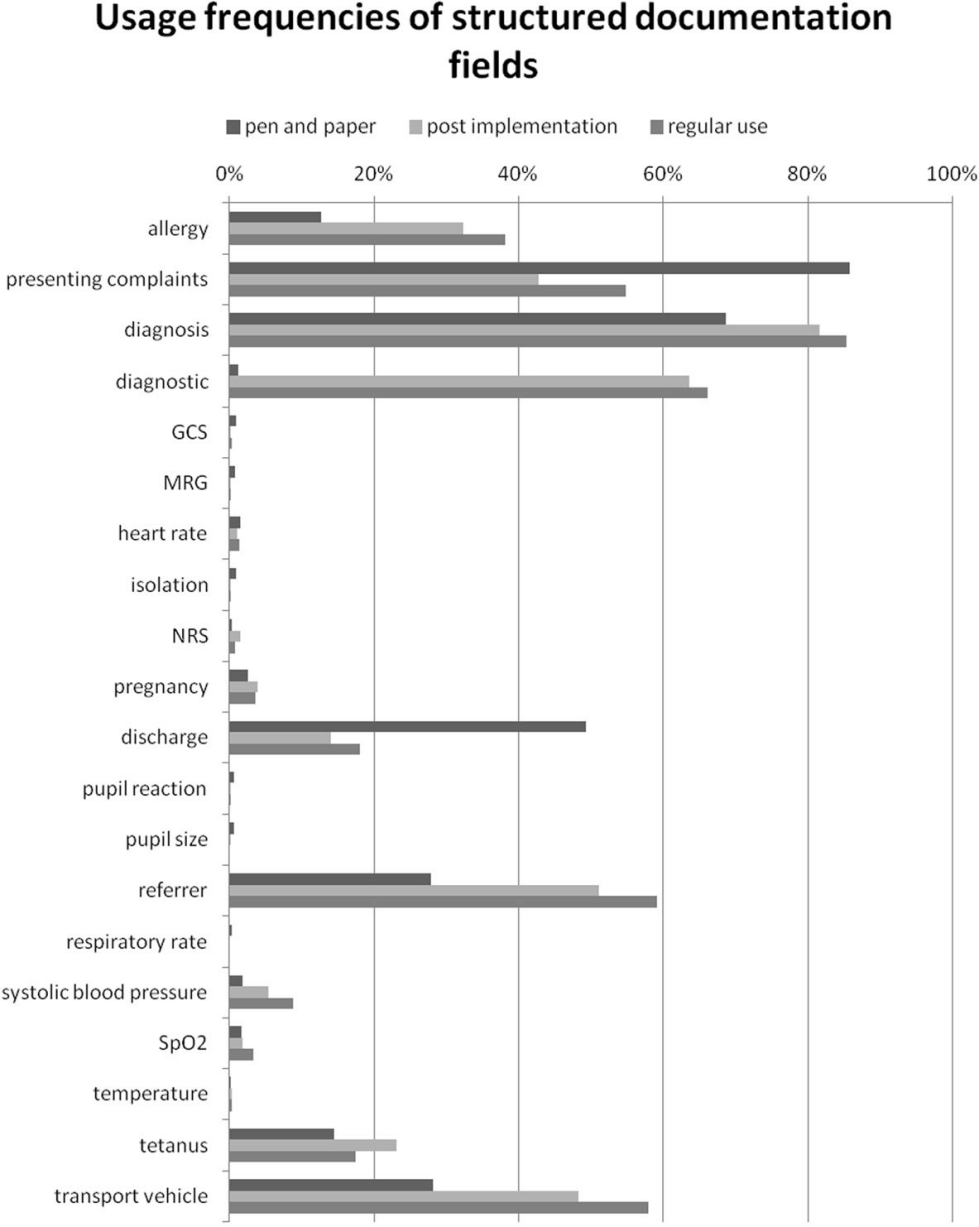
Usage frequencies of structured documentation fields. Comparison of usage of structured treatment information fields in relation to the documentation group. Availability increased significantly except in presenting complaints and discharge fields, χ^2^-test

Overall, the availability of structured information from pen-and-paper to PDMS documentation was increased (Fig. 4). The main improvements were seen in structured documentation of allergies, main diagnosis, diagnostic, referrer, vaccination status against tetanus, and transport vehicle. Here, a significant increase (*p* < 0.05) of documentation frequency could be proven. However, the documentation frequency of presenting complaints and discharge were significantly decreased in PDMS periods (*p* < 0.05; Fig. 4).

## Discussion

This is the first time that the complete GEDMR has been integrated into an existing timeline-based PDMS to adapt the system for use in the ED. Electronic implementation increased the availability of structured anamnesis and treatment information but also increased treatment time of traumatological patients.

There are various systems available for electronic documentation. We focused on a timeline-based system already in use at the hospital, which is especially useful for mapping the course of treatment. This offers advantages for time-consuming diagnostic tests or patients that require continuous monitoring. Furthermore, changes in conditions and impending emergency situations can be more easily recognized [15].

In general, electronic systems offer several advantages such as ubiquitous availability of patient documentation and optimized information management in order to improve the efficacy of communication [16]. In comparison to pen-and-paper systems, computerization improved legibility and enabled simultaneous, remote access [17]. Despite these advantages, the acceptance of electronic documentation systems by physicians remains very low [18, 19]. In our study a usage frequency of only 59.4% for the PDMS was reached in the regular-use period. There are various reasons for low acceptance, such as physicians’ complaints of a lack of training, doubts regarding data privacy, and increased time consumption for data input [18, 19]. As these reasons were mainly end user issues, the explicit reasons for low acceptance in our study could not ultimately be evaluated by our aggregated treatment and diagnostic documentation data. This should be addressed in a future study, such as an end-user survey including epidemiological data or prospective surveys capturing the reason for choosing pen-and-paper or electronic documentation.

Input time in electronic systems was indeed shown to be higher than that of paper-based documentation [20, 21]. Based on these findings, we can infer an increase in treatment time after implementation of electronic documentation. In the field of trauma surgery, treatment times are an important factor, since the number of patients is high. Therefore, increased treatment times are a likely factor for low acceptance of electronic documentation. Limiting our results, however, an explicit reason for the increase in treatment time could not be proven by the study design. As several factors seem to be possible, such as increased documentation time or increase in treatment time triggered by workflow support of the EDIS, this should be addressed in a second step by targeted end-user surveys.

It has already been shown that electronic documentation triggered increases in treatment times that resulted in a shift in hospital workflow [20, 21]. On the other hand, while electronic systems increase initial documentation times, they also do increase the legibility and availability of data, which can reduce time consumption for subsequent treatments [20].

Other potential barriers to acceptance and input performance are systems with high complexity and a confusing user interface [22], which in the worst case scenario reduces end users’ productivity and efficacy [21]. Consequently, improved system clarity is a main target for optimizing input and training time. To reduce the complexity of our system we distributed the 796 data items of the GEDMR across 33 dialog windows adapted to the regular workflow of the ED. These dialog windows could be loaded on demand, making the complexity of the electronic patient chart adaptable to the treatment case. Regarding the usage frequency of only 59.5% for the PDMS in regular-use period, one of the reasons for low acceptance in our study could be system complexity. However, it was shown that the gap between IT and physicians remained large [23]. Therefore, the implementation team should be interdisciplinary and should also include specialists in IT to address this problem [24, 25]. Despite its advantages, electronic systems also have a number of risks. After implementation of one electronic system in a pediatric clinic, a significant increase in mortality was observed [1, 26]. This effect was argued to be the result of a significant change in workflow and reduction of direct communication between physicians and nurses [1, 26, 27]. Overall, potential negative effects include workflow problems, migration problems from paper-based to electronic documentation, changes in communication patterns and practices, dependency on the electronic system, etc. [1, 28]. To minimize these effects, the implementation of our PDMS was performed by a physician working regularly in the ED and knowing the workflows and communication patterns of the particular ED.

In addition to regular clinical work, electronic systems also offer advantages for secondary use of the data such as benchmarking, quality management, surveillance and health services research. Electronic documentation and database storage simplify these processes significantly because of improved availability and the possibility of automated data processing in comparison to paper-based documentation. Moreover, harmonization via the GEDMR is important to ensure comparability between different EDs regarding health services research.

Another major advantage of electronical documentation systems is the ability to continuous data processing [17]. Automated export of treatment data in medical registries can be implemented, leading to data availability for research without any extra work for a physician or a study nurse.

The AKTIN project has implemented the GEDMR in order to establish a national ED data registry [29]. The purpose of this registry is reusing medical ED documentation, without further user interaction, for health services research and surveillance by using health information exchange technology for export into a registry. A corresponding interface for the automated export of the data from the ED to the registry has been implemented in 2017 and is used for first data queries.

## Conclusions

Implementation of the GEDMR in the PDMS ICUdata resulted in a usage frequency of 59.4% of the electronic health record. Availability of structured treatment data was improved. However, the treatment time was also increased by 29 min in the post-implementation period and 35 min in the regular-use period compared to the pen-and-paper group. Further work is necessary to evaluate the reasons for increased input time and low acceptance. Regarding the present results, targeted actions could be taken to increase acceptance and decrease input time or verify that increased time was due to medically-justifiable events that may have been missed in the paper system. Taken together, implementation of standardized documentation protocols into electronical documentation systems can serve as the basis for automated data export enabling benchmarking and quality management systems.

## Data Availability

The datasets generated and/or analysed during the current study are not publicly available due data privacy rules but are available from the corresponding author on reasonable request.

## Abbreviations

AKTIN: National Emergency Department Register: Improvement of Health Services Research in Acute Medicine in Germany
ED: Emergency department
EDIS: Electronic emergency department information system
GEDMR: German Emergency Department Medical Record
HIS: Hospital information system
HL7-CDA: HL7 Clinical Document Architecture
ICU: Intensive care unit
PDMS: Patient data management system
SEM: Standard error of means
